# Electrospun Scaffolds for Corneal Tissue Engineering: A Review

**DOI:** 10.3390/ma9080614

**Published:** 2016-07-27

**Authors:** Bin Kong, Shengli Mi

**Affiliations:** 1 Biomanufacturing Engineering Laboratory, Graduate School at Shenzhen, Tsinghua University, Shenzhen 518055, China; lingxue0313@163.com; 2 Macromolecular Platforms for Translational Medicine and Bio-Manufacturing Laboratory, Tsinghua-Berkeley Shenzhen Insititute, Shenzhen 518055, China; 3 Open FIESTA Center, Tsinghua University, Shenzhen 518055, China

**Keywords:** corneal tissue, electrospinning, nanofibrous scaffold, polymer

## Abstract

Corneal diseases constitute the second leading cause of vision loss and affect more than 10 million people globally. As there is a severe shortage of fresh donated corneas and an unknown risk of immune rejection with traditional heterografts, it is very important and urgent to construct a corneal equivalent to replace pathologic corneal tissue. Corneal tissue engineering has emerged as a practical strategy to develop corneal tissue substitutes, and the design of a scaffold with mechanical properties and transparency similar to that of natural cornea is paramount for the regeneration of corneal tissues. Nanofibrous scaffolds produced by electrospinning have high surface area–to-volume ratios and porosity that simulate the structure of protein fibers in native extra cellular matrix (ECM). The versatilities of electrospinning of polymer components, fiber structures, and functionalization have made the fabrication of nanofibrous scaffolds with suitable mechanical strength, transparency and biological properties for corneal tissue engineering feasible. In this paper, we review the recent developments of electrospun scaffolds for engineering corneal tissues, mainly including electrospun materials (single and blended polymers), fiber structures (isotropic or anisotropic), functionalization (improved mechanical properties and transparency), applications (corneal cell survival, maintenance of phenotype and formation of corneal tissue) and future development perspectives.

## 1. Introduction

The cornea is a transparent, avascular, multi-laminar structure that forms a barrier to protect the intraocular structure and microenvironment while refracting light onto the retina [1,2,3] and it is one of the most important tissues involved in vision [4]. The cornea consists of five distinct layers: the corneal epithelium (outermost layer), Bowman’s layer, the stroma, Descement’s membrane and the corneal endothelium (innermost layer) [1,5]. The thickness of the human cornea is approximately 500 µm, and the stroma with its keratocytes and aligned collagen fibers makes up the main part of the cornea [6]. The epithelium is composed of epithelial cells that can be approximately five to seven layers in thickness [5]. However, due to corneal trauma and ulceration, bacterial and viral infections and heritable conditions, corneal diseases constitute the second leading cause of vision loss and affect more than 10 million people globally [7,8,9,10]. Grafting allogenic corneal tissue is one of the primary therapies for serious diseases of the cornea because of its accessibility and immune privilege. However, there is a severe shortage of donor corneal tissue worldwide. Thus, it is very important and urgent to construct corneal equivalents to replace pathologic corneal tissue, and developments in tissue engineering make this possible [11,12,13]. One way is to use human amniotic membrane (HAM), which is the most widely used substrate for the construction of damaged ocular surfaces and has been considered a gold standard scaffold for epithelial cell expansion [14,15,16]. HAM possesses the ability to reduce scarring and inflammation, to enhance wound healing, and to provide anti-fibrotic effects, yet this membrane is associated with some drawbacks, including risks of contamination and transmission of infectious diseases and biologic variability between donor tissues [17,18,19]. Another method is the use of a fully bioengineered cornea with high biocompatibility and superior biological performance. The major difficulty in producing such a construct has been the generation of a corneal equivalent in vitro that exhibits strength and transparency equivalent to those of native tissue [2]. There are a variety of approaches that have been explored to construct three-dimensional (3-D) bioengineered corneal tissues in vitro. These strategies are to design biomimetic matrix systems for corneal tissue construction, such as via the hydrogel technique [20,21], prefabricated matrices [22,23], and decellularized corneal tissues [24,25,26]. Great potential has been demonstrated with different systems, such as nano-scale modification of porous gelatin materials with chondroitin sulfate using carbodiimide chemistry to construct corneal stromal tissue, functionalized corneal tissues by highly decellularized corneal tissue. However, these systems also have several limitations, such as insufficient mechanical strength and suturability of the hydrogel system, leading to further cross-linking, low production and immunogenicity of decellularized systems. Prefabricated matrices (e.g., nanofibrous and microporous scaffolds) have offered a feasible strategy to address these challenges in corneal tissue engineering. Particularly, nanofibrous scaffolds fabricated using the electrospinning technique have been increasingly explored.

Electrospinning is a versatile fabrication process that uses a high voltage between a syringe and a deposition target (or collector) to draw nano- or micro-scale fibers from the material dispensed by the syringe [27]. Electrospinning equipment is very simple, comprising a high-voltage direct current power supply (5 to 50 kV), a spinneret (typically a hypodermic syringe needle) connected to a high-voltage power supply, and a grounded collector. The theory of this technology is that under an electrostatic field, when the repulsive force between charged particles in polymer solutions overcomes the solution surface tension, the droplet that is suspended on the top of the pipe will form a charged jet, and after repeatedly splitting in the electrostatic field and the evaporation of the solvent, fibers finally form in the collector. The deposition of these fibers in a specifically located collector permits the generation of three-dimensional (3D) fibrous scaffolds. To date, more than 200 polymers have been successfully electrospun to nano-scale or micro-scale fibers, including natural materials (e.g., collagen, gelatin, hyaluronate (HA), chitosan, silk fibroin (SF), etc.) and synthetic materials (e.g., polycaprolactone (PCL), poly-l-lactic acid (PLLA), poly(lactide-*co*-glycolide) (PLGA), polyethylene oxide (PEO), etc.) [23,28,29,30,31,32,33,34]. There are a number of processing parameters that can affect the morphology of the fibers, which include the chemical nature of the polymer solution (i.e., molecular weight, concentration, and solvent), the applied voltage, the distance between the spinneret tip and the collector, the feeding rate, the capillary diameter, the humidity and the temperature. Based on the applied parameters, the morphology can be varied to produce uniform fibers, beaded fibers, or fibers with spindles on a string [35]. Furthermore, several authors have reported studies on the use of patterned collectors to achieve different architectures of the electrospun scaffolds using either rotating or static collectors for fiber deposition [36,37]. The electrospun products have a wide variety of applications, such as in micro-reactors and sensors or for filtration, energy storage, catalysis, biomedicine and tissue engineering applications [38,39,40,41].

Recently, electrospinning has attracted increased interest for fabricating biomimetic engineering functional corneal tissue due to the close structural resemblance of the constructs to native ECM and its high surface area–to-volume ratio and good porosity, which provide support for cell adhesion and movement, proliferation and differentiation, as well as excellent mechanical properties, easy manipulation of fiber properties, great material handling, suturability for implantation, and scalable production [28,42,43]. In this review, we aim to present an overview of recent studies of electrospun scaffolds with a focus on their applications in corneal tissue engineering. First, we present various research methods to fabricate scaffolds with single or blended components, isotropic or anisotropic structures, and improved transparency and mechanical properties, which all have shown potential for improving the performance of the design and manipulation of nanofibrous scaffolds for corneal tissue regeneration. Then, we discuss the current applications of these electrospun scaffolds for the construction of functional corneal tissues, with a focus on the design and manipulation of nanofibrous scaffolds for corneal tissue regeneration. Finally, the challenges and future perspectives for the development of electrospun materials for engineering functional corneal tissues are also addressed.

## 2. Materials of Electrospun Scaffolds

As mentioned above, over 200 polymers have been successfully electrospun to nano-scale or micro-scale fibers. However, only those with excellent biocompatibility have been widely utilized for tissue engineering applications. In addition to biocompatibility, electrospun scaffolds also need to offer additional properties required for corneal tissue engineering, including a degradation rate that is comparable to the regeneration rate of native ECM to provide sustained support for corneal tissue regeneration, mechanical properties that match those of the human cornea (elongation at break and tensile strength are up to ≈0.19 and ≈3–5 MPa, respectively), and improved scaffold transparency for inducing visual function of the engineered corneal tissues. Table 1 lists all the polymers that have been fabricated into electrospun scaffolds and explored for corneal tissue engineering.

According to Table 1, the most commonly used materials for electrospinning in corneal tissue engineering include single polymers and blended polymers. One material can dissolve in multiple solvents or blended solvents of two or more components. The properties of the polymer solution have an essential effect on the morphology of the electrospun fibers, such as the solution concentration and molecular weight. In general, when the other parameters are consistent, the diameter of the electrospun fibers is positively correlated with the solution concentration. An improper concentration may lead to the formation of beads or the inability to form fibers. Through the control of the electrospun collector or the electrostatic field, scaffolds with various fiber arrangements can be easily and conveniently obtained to simulate the structure of the ECM in natural corneal tissue (e.g., aligned fibers in the corneal stromal layer). The material components, fiber morphology and arrangement, the surface chemical modification of the scaffold (e.g., plasma) and the information molecules (e.g., epidermal growth factor) can affect the function of electrospun scaffolds and, through the regulation of these factors, scaffolds with excellent properties (e.g., biological, mechanical properties and high transparency) can be fabricated, which can improve the adhesion, movement, proliferation and differentiation of corneal cells and further the formation of corneal tissue.

### 2.1. Single Electrospun Scaffolds for Corneal Tissue Engineering

**Figure 1:**
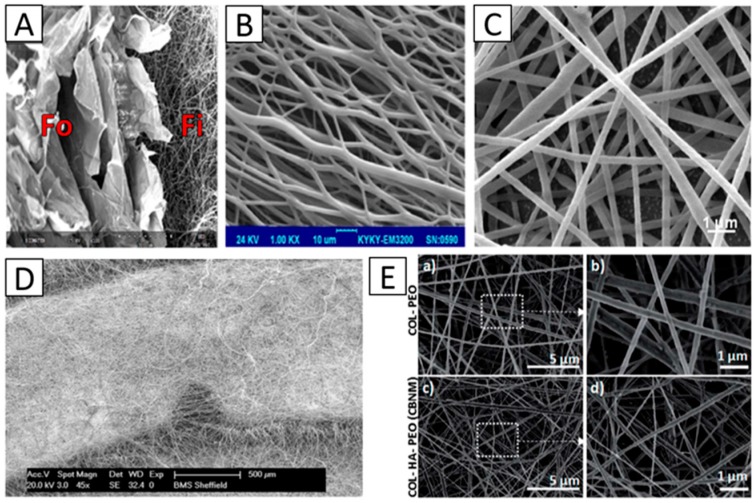
Scanning electron micrographs of the scaffolds. (**A**) Cross-section (foam layer on the left and fibers on the right). Reproduced from [48], with permission from © 2014 Tylor and Francis; (**B**) Oriented nanofibers of silk scaffold. Reproduced from [51], with permission from © 2015 Tylor and Francis; (**C**) Randomly oriented PCL scaffold. Reproduced from [44], with permission from © 2014 The Association for Research in Vision and Ophthalmology; (**D**) A section of the electrospun scaffold showing a horseshoe electrospun micro-pocket. Reproduced from [27], with permission from © 2012 Elsevier; (**E**) Electrospun nanofibrous membranes with binary COL-PEO (**a** and **b**) and ternary COL-HA-PEO compositions (**c** and **d**). Reproduced from [43], with permission from © 2014 The Royal Society of Chemistry.

Natural materials, such as collagen, silk and gelatin, which have excellent biocompatibility, biodegradability and low immunogenicity, have been extensively utilized for corneal tissue engineering. Donna Phu, B.S. et al. electrospun type I collagen scaffolds for culturing corneal fibroblasts ex vivo that mimicked the microenvironment in the native cornea, and they recently investigated the effect of the scaffold nanostructure and composition on the phenotype of corneal stromal cells [49]. Lindsay, et al. also electrospun collagen type I fibers that replicated the unique morphology and arrangement of collagen type I fibers in the native cornea [50]. Collagen-chondroitin sulfate foam coated with a collagen electrospun mat was constructed by A. Acun to mimic the stromal layer and Bowman’s layer (Figure 1A). The stromal layer substitute was made of *N*-ethyl-*N*-(3-dimethylaminopropyl) carbodiimide/*N*-hydroxysuccinimide-cross-linked collagen–chondroitin sulfate foam and seeded it with primary human corneal keratocytes. Retinal pigment epithelium (RPE) cells served as the epithelial layer after seeding on a dehydrothermally cross-linked collagen type I fibrous mat deposited directly on top of the foams by electrospinning. Physical characterizations and in vitro studies showed that the designed cornea replacement was suitable for cell attachment and growth, and co-culture of the two cell types induced more ECM deposition than single cell–seeded constructs [48]. An silk electrospun mat (Figure 1B), which was highly compatible, was produced to serve as a potential alternative substrate to HAM. Human limbal stem cells could favorably attach and proliferate on the nanofibrous surface, and cells were able to infiltrate the nanofibers and successfully form a 3D corneal epithelium [51].

Although natural materials have many excellent biological properties, their poor mechanical properties will limit their clinical applications to a large extent. Synthetic polymers, especially FDA-approved polymers, such as PLGA, PCL, and PLA (poly lactic acid), have been used in corneal tissue engineering due to their superb mechanical properties. PCL has been electrospun into nanofibrous scaffolds (Figure 1C), allowing for inoculation of limbal epithelial cells [14], human corneal epithelial cells [44], rabbit keratocytes [45] and rabbit limbal stem cells [46] on the surface of the scaffold. Studies showed that the PCL scaffold has good biocompatibility and can improve cell attachment and proliferation. Ílida Ortega et al. combined an electrospun PLGA mat with microstereolithography for the fabrication of corneal membranes that mimic, to a certain extent, the limbus (Figure 1D). Specifically, they used polymeric structures produced by microstereolithography as micro-fabricated collectors for electrospinning. The deposition of PLGA on these collectors then achieved the generation of electrospun patterned scaffolds for corneal regeneration in a one-stage procedure [27].

### 2.2. Blended Electrospun Scaffolds for Corneal Tissue Engineering

A blended electrospun scaffold can be fabricated using a polymer blend (Figure 1), aiming to regulate the mechanical, chemical and biological properties of the scaffolds to promote corneal tissue regeneration. Generally, blended electrospun scaffolds are made of natural and synthetic polymer blends, which can combine the excellent biocompatibility of natural polymers and the great mechanical properties of synthetic polymers. For instance, gelatin has been frequently used to fabricate electrospun scaffolds for engineering corneal tissues; however, such scaffolds have limited clinical applications due to their insufficient mechanical strength (≈0.1 MPa) and fast degradation rate (water-soluble) [57]. Although further cross-linking treatments can slightly improve the mechanical strength of scaffolds, this has raised another concern of using a non-biocompatible cross-linker (i.e., glutaraldehyde) [58,59]. To overcome these challenges, poly (3-hydroxybutyrate-co-3-hydroxyvalerate) (PHBV)/gelatin [42], collagen/HA/PEO (Figure 1E) [43], gelatin/PLLA [54,55] and SF/P (LLA-CL) [56] have been electrospun.

In addition to adjusting different polymer combinations, the ratio of polymer blends can also be changed to regulate the mechanical properties and the degradation rate to match natural ECM. For example, poly(glycerol sebacate) (PGS)/PCL nanofibrous scaffolds were fabricated by electrospinning different weight ratios of PGS and PCL (1:1, 2:1, 3:1, and 4:1) and the Young’s moduli decreased with the increasing PGS content. The Young's modulus of the 4:1 blended scaffold was determined to be 1–1.2 MPa, fitting nicely in the range of the mechanical properties of the native stroma [3,53]. SF/P (LLA-CL) scaffolds were fabricated by electrospinning different blended ratios (100:0, 75:25, 50:50, 25:75, and 0:100). A tensile test showed that when the ratio was 50:50, the tensile strength of the scaffold was most similar to the native corneal tissue [56]. Blended nanofibrous scaffolds using more than two polymers have also been electrospun to achieve better control of the properties of the scaffolds or to achieve additional functions for corneal tissue engineering applications [60,61]. For instance, collagen/HA/PEO scaffolds were fabricated through electrospinning and exhibited excellent biocompatibility and mechanical properties and the ability to promote cell attachment and corneal epithelium regeneration [43]. These improvements are primarily attributed to the close similarities of the scaffolds to the native ECM in human cornea, including mechanical strength and biological functions.

## 3. Methods of Fabrication of Electrospun Scaffolds

In this part, strategies for the fabrication of electrospun scaffolds that could be potentially utilized for corneal tissue engineering are detailed.

### 3.1. Electrospun Scaffolds with Isotropic or Anisotropic Structure

The control over the structural properties of electrospun scaffolds (e.g., fiber morphology, fiber diameter, and fiber orientation) has been demonstrated as an important factor in improving their performance for tissue engineering applications. By utilizing special collectors, aligned scaffold fibers can be obtained. It is well known that cells react differently to micro-topography and nano-topography, and mimicking natural tissues has been attempted by cell guidance using physical cues [62,63]. These studies have suggested that some cells can distribute randomly within a non-woven scaffold while growing orderly on an aligned scaffold.

#### 3.1.1. Electrospun Scaffolds with Randomly Oriented Fibers

Chaotic fibers can be easily obtained using a traditional collector, such as a cylinder or a metal mesh. Pallavi Deshpande et al. electrospun chaotic PLGA mats to provide a biodegradable cell carrier system for limbal epithelial cells. The study showed that the limbal cells formed a continuous multilayer of cells on either side of the scaffold. Scaffolds of cells showed signs of the onset of degradation within two weeks in culture media at 37 °C. They suggested that this chaotic electrospun mat could be used as a replacement for the HAM in the treatment of limbal stem cell deficiency, lowering the risk of disease transmission to patients [47] (Figure 3A,B). Jing Yan et al. produced randomly oriented gelatin/PLLA nanofibrous scaffolds and inoculated corneal epithelial cells on the surface of the scaffolds. The result demonstrated that the corneal epithelial cells grew well on the randomly oriented scaffolds, which was favorable for the reconstruction of corneal epithelium in corneal tissue engineering [55].

#### 3.1.2. Electrospun Scaffolds with Aligned Fibers

**Figure 2:**
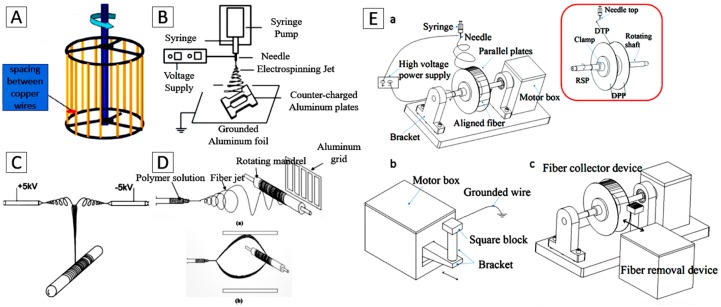
The electrospinning collector for the aligned nanofibers. (**A**) A rotating copper wire drum. Reproduced from [55], with permission from © 2015 The Royal Society of Chemistry; (**B**) Auxiliary electrode/electrical field. Reproduced from [72], with permission from © 2007 American Chemical Society; (**C**) Two spinnerets with opposite voltages and directions. Reproduced from [73], with permission from © 2006 Elsevier; (**D**) Frame collector. Reproduced from [74], with permission from © 2003 Elsevier; (**E**) Parallel double-thin plates collector. Reproduced from [75], with permission from © 2015 Elsevier.

For certain applications in tissue engineering, scaffolds with aligned fibers possess unique electrical, optical, and mechanical properties and are often more desirable to guide cell growth with the desired anisotropy [64,65,66,67,68]. Several fiber collection methods, including the use of cylinders with high rotational speed [36], wire drum collectors (Figure 2A) [55,69], auxiliary electrode/electrical fields (Figure 2B) [70,71,72], two spinnerets with opposite voltages and directions (Figure 2C) [73], frame collectors (Figure 2D) [74] and parallel double-thin plate collectors (Figure 2E) [75], have been developed to align the fibers on the collector.

**Figure 3:**
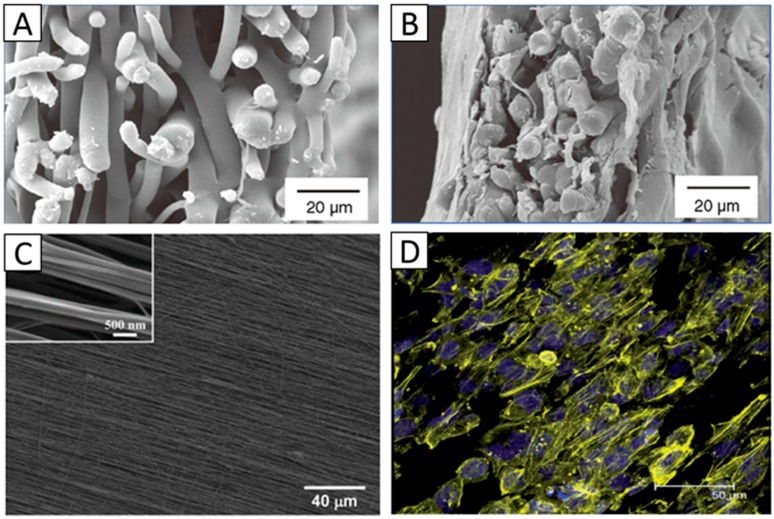
SEM images of randomly aligned PLGA scaffolds: (**A**) Cross-sectional view of cell-free scaffolds; (**B**) Cross-sectional view of scaffolds cultured with limbal epithelial cells for 14 days. Panels (**A**,**B**) Reproduced from [47], With permission from © 2010 Future Medicine; (**C**) SEM image of aligned fibrous PEUU scaffold. Reproduced from [8], with permission from © 2012 Elsevier; (**D**) Stained human corneal epithelial cells with DAPI (blue, nuclei) and phalloidin (yellow, F-actin) after three days of culturing on the aligned PGS/PCL nanofibrous scaffolds. Reproduced from [53], with permission from © 2014, The Royal Society of Chemistry.

The aligned fibers mimicking the parallel orientation of native tissues have demonstrated favorable cell adhesion, migration and proliferation for corneal, cardiac, neural, and skeletal muscular tissues [64,76]. For native corneal stroma, which consists of aligned collagen fibers, the aligned feature is critical for mechanical and transparency properties, which is important for physiological functions. The control and maintenance of the keratocyte phenotype is vital for developing in vitro tissue-engineered strategies for corneal repair. Studies have demonstrated that scaffold nanostructures have a great influence on the phenotype of keratocytes. Donna Phu, B.S et al. electrospun aligned and unaligned collagen nanofibrous scaffolds, and rabbit-derived corneal fibroblasts were cultured on two scaffolds and assessed for expression of a-smooth muscle actin, a protein marker upregulated in hazy corneas. The result showed that cells grown on aligned collagen type I fibers exhibited significantly greater down-regulated a-smooth muscle actin protein expression than unaligned collagen scaffolds [49]. Samantha L. Wilson et al. electrospun multiple orthogonal aligned poly (L, D lactic acid) (PLDLA) scaffolds, inoculating human corneal stromal cells on the surfaces of the scaffolds. The matrix elasticity (elastic modulus) and the dimensional changes were indicative of changes in cell phenotype from contractile fibroblasts to quiescent keratocytes [1]. These studies all demonstrated that aligned fibrous structures are favorable for reverting corneal fibroblasts to a keratocyte phenotype in a 3D construct. Jian Wu et al. electrospun aligned poly(ester urethane) (PEUU) mats, inducing alignment of cultured human corneal stromal stem cells (hCSSCs), which elaborated a dense aligned collagenous matrix, 8–10 µm in thickness, deposited on the PEUU substratum. This matrix contained collagen fibrils of uniform diameter and regular interfibrillar spacing, exhibiting global parallel alignment similar to that of native stroma [8] (Figure 3C). Jing Yan et al. electrospun aligned and unaligned gelatin/PLLA scaffolds, and the result showed that the aligned scaffold exhibited a higher tensile modulus, a higher break strength, and a lower elongation at break than randomly oriented scaffold and that keratocytes were interacting more favorably on the aligned scaffold [55]. Aligned PGS/PCL scaffolds were also electrospun and induced the aligned growth of corneal epithelial cells on the scaffolds [53] (Figure 3D). All these studies indicate that aligned electrospun scaffolds can be ideal matrices for guiding corneal cells into organized tissues that closely mimic the native corneal tissue.

### 3.2. Functionalization of Electrospun Scaffolds

In addition to the microstructure of electrospun scaffolds, transparency and mechanical properties are essential characteristics when constructing corneal substitutes in vitro.

#### 3.2.1. Electrospun Scaffolds with Improved Transparency

Optical transparency is an important property that should be considered while developing a bioengineered corneal construct. A healthy cornea is required for clear vision and it contributes two-thirds of the total refractive power of the eye, which is the most remarkable property of the cornea. For this reason, a corneal equivalent fabricated by tissue engineering should be able to transmit most of the visible light to mimic the natural behavior of the native cornea.

Many polymers can be electrospun into nanofibrous scaffolds, including natural and synthetic materials. However, when applied in corneal tissue engineering, not all these materials are suitable because of their opacity or low transparency. There are three primary methods to improve the transparency of electrospun mats. One is post-modification of the scaffold via plasma discharge treatment. Additionally, plasma treatment further enhances the cell adhesion properties of these scaffolds. Surface modification by plasma treatment is a well-established method for modifying surface chemistry without changing morphology in an eco-friendly way [77]. Plasma is a partially ionized gas that contains a mixture of ions, electrons, neutral molecules, and free radicals that are able to create active species on a plasma-treated surface. Shweta Sharma et al. electrospun PCL nanofibrous scaffold subjected to helium-oxygen (He/O_2_) plasma treatment. The results indicated that a plasma-treated stromal equivalent can transmit 37% more light than the untreated plasma stromal equivalent [44]. Haleh Bakhshandeh et al. fabricated a two-part artificial cornea as a substitute for penetrating keratoplasty in patients with corneal blindness. The peripheral part of the artificial cornea consisted of plasma-treated electrospun PCL nanofibers, which were attached to a hydrogel disc of polyvinyl alcohol (PVA) as a central optical part. The result also showed a high transparency with 85% light transmittance when measured in the 400–800 nm wavelength range, which is very similar to that of the natural cornea [46].

**Figure 4:**
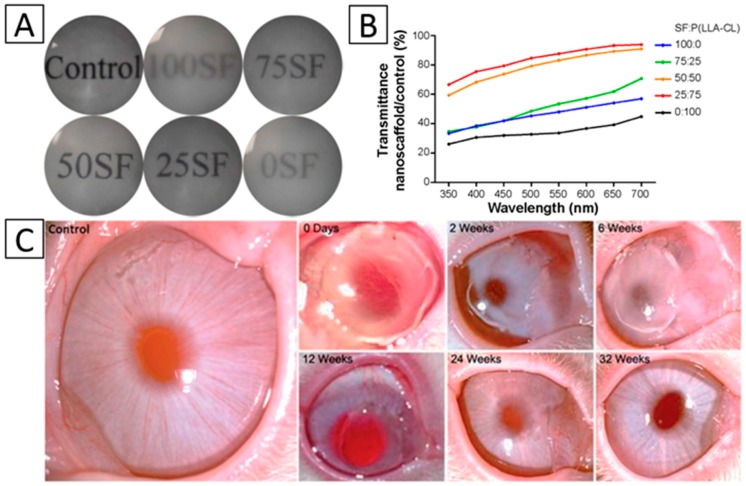
Transmission of light in nanofibrous membranes. (**A**) General transmission of light with different blend ratios; (**B**) Accurate transmission percentage compared with the control group of A. Panels (**A**,**B**), Reproduced from [56], with permission from © 2015 Chen et al; (**C**) Restorative process of the corneal transparency of NZWRs during a 32-week post-operative slit-lamp examination. Reproduced from [55], with permission from © 2015 The Royal Society of Chemistry.

The second method is through the blending of different materials, generally natural and synthetic polymers. Juan Ye et al. produced collagen/HA/PEO electrospun mat coatings with chitosan on the surface. The result showed that the scaffold had an excellent transparency [43]. Chen, et al. electrospun SF/P (LLA-CL) nanofibrous scaffolds, and they studied the effect of blended ratios (100:0, 75:25, 50:50, 25:75, and 0:100) on the transparency of the scaffold. The result demonstrated that the 25:75 blended ratio SF/P (LLA-CL) scaffold had the best transmittance [56] (Figure 4A,B). PLLA/gelatin nanofibrous scaffolds were electrospun by Zhang et al., and the blended material also exhibited high transparency [55] (Figure 4C).

Notably, keratocytes usually stay in the quiescent state and maintain non-crystalline structures to make the corneal transparent and optimal refraction. Recent studies have identified the importance of the intracellular protein expression of keratocytes in maintaining corneal transparency [78]. The keratocytes in the quiescent phenotype express two characteristic proteins: transketolase (TKT) and aldehyde dehydrogenase class 1A1 (ALDH1A1) [79]. A decrease in the expressions of TKT and ALDH1A1 in keratocytes leads to a marked decrease in corneal transparency and increased light scattering from keratocytes [80]. Thus, the third method is to keep keratocytes maintaining the quiescent phenotype. Samantha L. Wilson et al. studied the influence of topographical and chemical cues on the phenotypical behavior of adult human-derived corneal stromal cells in 3D multi-layered organized PLDLA electrospun constructs. The results indicate that the synergistic effect of nanofibers and serum-free media plus insulin supplementation provide the most suitable topographical and chemical environment for reverting corneal fibroblasts to a keratocyte phenotype in a 3D construct [1].

#### 3.2.2. Electrospun Scaffolds with Improved Mechanical Properties

Not only do the scaffolds need to provide high transparency and suitable refractive power, but they must be able to carry the tension induced from high intraocular pressure and eye movements [3]. The scaffold should therefore have similar mechanical properties to natural corneal tissue.

**Figure 5:**
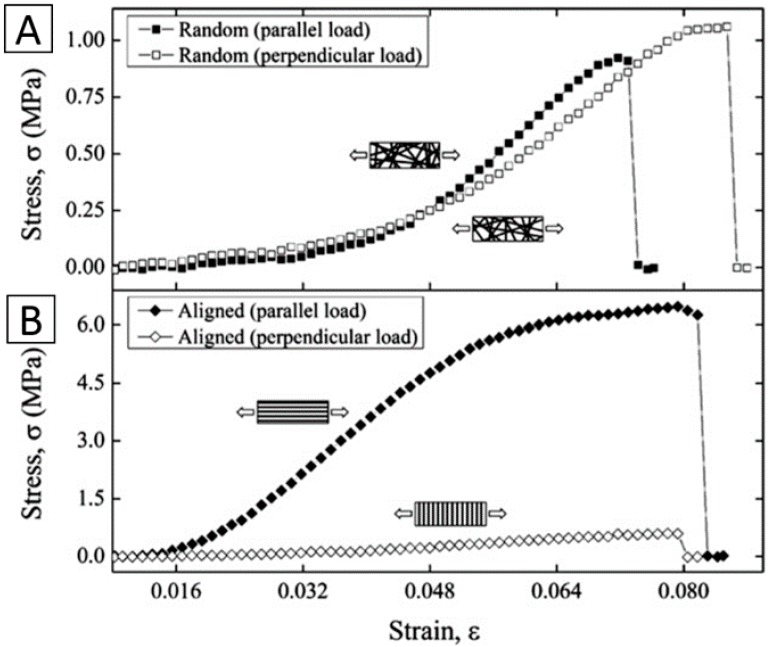
A comparison of stress-strain curves of (**A**) randomly oriented and (**B**) aligned electrospun gelatin mats under two different loading orientations. Reproduced from [52], with permission from © 2013 Elsevier.

Haleh Bakhshandeh et al. electrospun PCL nanofibrous scaffolds post-treated with plasma to improve their mechanical properties. The result demonstrated that the Young’s modulus value of the electrospun PCL skirt was 7.5 MPa, which is in line with the elasticity range of natural human corneas (0.3–7 MPa) [46]. Electrospun gelatin nanofibers were infiltrated with alginate hydrogels, yielding transparent, mechanically reinforced hydrogels by Khaow Tonsomboon. The electrospun gelatin nanofibers improved the tensile elastic modulus of the hydrogels from 78 ± 19 kPa to 450 ± 100 kPa. The developed fiber-reinforced hydrogels showed great promise as mechanically robust scaffolds for corneal tissue engineering applications [52] (Figure 5). S. Salehi et al. electrospun aligned nanofibers of PGS/PCL and studied the effect of the blended ratio on the mechanical properties. The elastic modulus of the fibers was found to decrease with the increased PGS/PCL blend ratios. In contrast, the surface modulus of the nanofibers, measured by nano-indentation, exceeded the elastic modulus by two orders of magnitude and increased with the weight ratio of PGS [53]. Juan Ye presented a versatile method utilizing electrospinning and surface modification processes to develop microstructurally stable (>20 MPa in tensile strength in the wet state) biomimetic nanofibrous collagen/HA/PEO membranes [43]. Jing Yan et al. constructed gelatin/PLLA nanofibrous scaffold by electrospinning, and they studied the effect of the alignment degree of fibers on the mechanical properties. Tensile tests of wet scaffolds indicated that the aligned scaffold exhibited a higher tensile modulus, higher break strength, and lower elongation at break than randomly oriented scaffolds [54].

## 4. Applications of Electrospun Scaffolds for Corneal Tissue Regeneration

### 4.1. Cell Survival and Differentiation of Corneal Cells

Scaffolds for corneal tissue engineering should support cell survival and promote cell differentiation toward the corneal phenotype, which can be achieved by electrospun scaffolds due their bio-mimicking nanofibrous structure and excellent biocompatibility [52,81].

**Figure 6:**
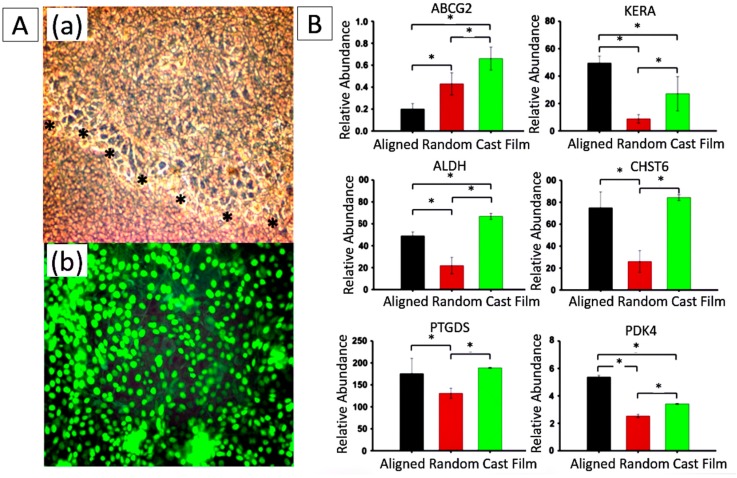
(**A**) Biocompatibility assessment of electrospun PCL nanofibers. (**a**) Phase contrast picture shows migration of human corneal epithelial cells over nanofibers (black stars line); (**b**) Epithelial cell sheet demonstrates high viability ratio of human corneal epithelial cells on nanofibers by their positive green staining. Cells were observed at 200× magnification. Reproduced from [14], with permission from © 2011 Molecular Vision; (**B**) Changes in gene expression of hCSSCs seeded on aligned fibrous substrates (black), random fibrous substrates (red) and cast films (green). mRNA abundance was compared with hCSSCs cultured in SCGM. Ratios of abundance of each transcript between hCSSCs seeded on different substrates cultured in keratocyte differentiation medium and in SCGM are expressed on a linear scale. Since KERA has no expression in hCSSCs cultured in SCGM, it is expressed in an absolute manner. Error bars show the SD of three independent samples. For each gene, expression levels were significantly different between the studied substrates (*p* < 0.05), except for CHST6 and PTGDS on aligned fibrous substrate and cast film. Reproduced from [8], with permission from © 2012 Elsevier.

Some electrospun scaffolds have also shown the ability to promote the differentiation of corneal stem cells into corneal epithelial cells or keratocytes. For example, Shweta Sharma at al. electrospun PCL nanofibrous scaffolds and inoculated limbal epithelial stem cells on their surfaces. The cells on the scaffold exhibited high bio-viability, and the expression of differentiation markers K3/12 suggests that cultivated limbal epithelial stem cells had the potential to differentiate into mature corneal epithelial cells [14] (Figure 6A). A substrate of aligned PEUU fibers produced by Samantha L. Wilson, inducing the alignment of cultured hCSSCs, elaborated a dense collagenous matrix deposited on the PEUU substratum. This matrix contained collagen fibrils with uniform diameters and regular interfibrillar spacing, exhibiting global parallel alignment similar to that of native stroma. The cells expressed high levels of gene products unique to keratocytes, including keratocan (KERA), aldehyde dehydrogenase 3A1 (ALDH), prostaglandin D2 synthase (PTGDS), corneal *N*-acetylglucosamine-6-*O*-sulfotransferase (CHST6) and pyruvate dehydrogenase kinase isoenzyme 4 (PDK4), indicating that the stem cells on the substrate differentiated into keratocytes [8] (Figure 6B).

### 4.2. Maintenance of Corneal Cell Phenotype

**Figure 7:**
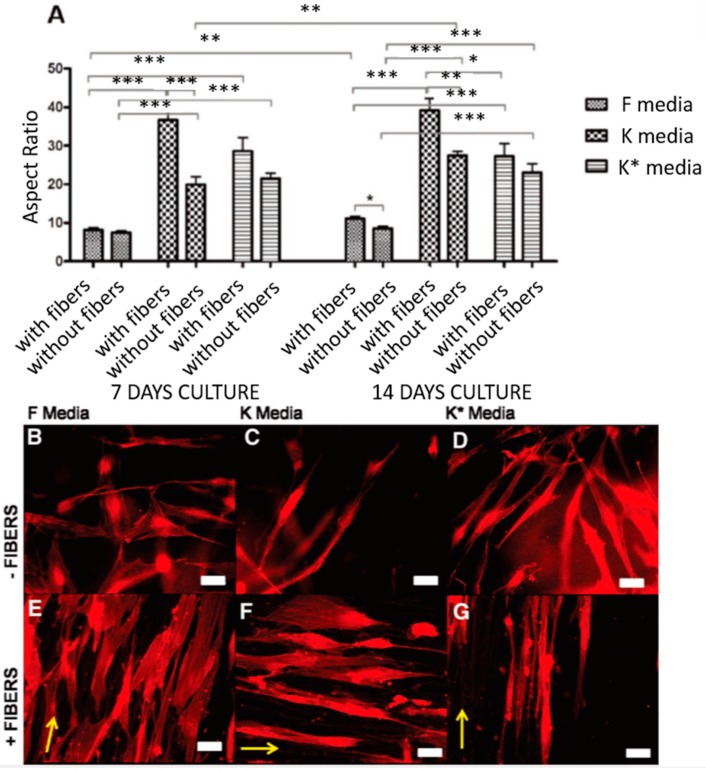
(**A**) Average aspect ratio for human-derived corneal stromal cells seeded in the hydrogel constructs with and without the inclusion of nanofibrous meshes cultured under F, K and K * media for seven and 14 days. * *p* ≤ 0.05, ** *p* ≤ 0.01, *** *p* ≤ 0.001. (**B**–**G**) are representative actin-stained cells of collagen hydrogel constructs with (**E**–**G**) and without (**B**–**D**) the incorporation of nanofibrous meshes following 14 days in culture in F, K and K * media, respectively. When cultured in fiber-free constructs, cells grown in serum-containing F media had a shorter, fusiform morphology (**B**) compared to the more elongated morphology of cells cultured in serum-free K and K * media (**C**,**D**). The addition of nanofibers increased organization within the constructs and encouraged all cells to align and adopt a more elongated morphology (**E**–**G**). Yellow arrows indicate the direction of fibers, scale bar = 50 μm. Reproduced from [1], with permission from © 2012 WILEY-VCH Verlag GmbH and Co. KGaA, Weinheim.

Control and maintenance of the corneal cell phenotype is vital for developing in vitro tissue-engineered strategies for corneal repair. Much research work has been performed to study how to maintain the phenotype of corneal cells, such as limbus stem cells, corneal epithelial cells and keratocytes. Limbus stem cells were demonstrated to retain a normal corneal stem cell phenotype on the electrospun silk scaffolds [51], PCL scaffolds [44,82] and blended PHBV/gelatin scaffolds [42]. The potentials of electrospun scaffolds made of polyhydroxybutyrate (PHB), PHBV, and PCL were studied to serve as drug-screening platforms for corneal keratocyte tissues by Pedram Azari et al. They cultured keratocytes on these scaffolds, and the result showed that cells had a high viability and proliferation and that PCL exhibited good potential for maintaining the cell phenotype with high expressions of LUM, ALDH, VIM, COL.1, and a-SMA2 [45]. Samantha L. Wilson studied the influence of topographical and chemical cues on the phenotypical behaviors of adult human-derived corneal stromal (AHDCS) cells in 3D multi-layered organized PLDLA electrospun constructs. The results indicated that the synergistic effect of nanofibers and serum-free media plus insulin supplementation provided the most suitable topographical and chemical environment for reverting corneal fibroblasts to a keratocyte phenotype in a 3D construct [1] (Figure 7). Donna Phu, B.S et al. investigated the effect of scaffold nanostructure and composition on the phenotype of corneal stromal cells. They cultured rabbit-derived corneal fibroblasts on aligned and unaligned collagen type I fibers. The results showed that cells grown on collagen scaffolds had reduced myofibroblast phenotype expression compared to cells grown on tissue culture plates. Cells grown on aligned collagen type I fibers exhibited significantly more down-regulated a-smooth muscle actin protein expression than unaligned collagen scaffolds [49].

### 4.3. Corneal Tissue Formation

As the cells cultured on electrospun scaffolds are capable of survival, differentiation and maintaining their phenotype, further attention should be paid to inducing the formation of corneal tissues. Because it is difficult to construct full-thickness corneal equivalents that include all five layers, most researchers have focused on the fabrication of one layer or two layers, such as the corneal epithelial layer or the stromal layer.

**Figure 8:**
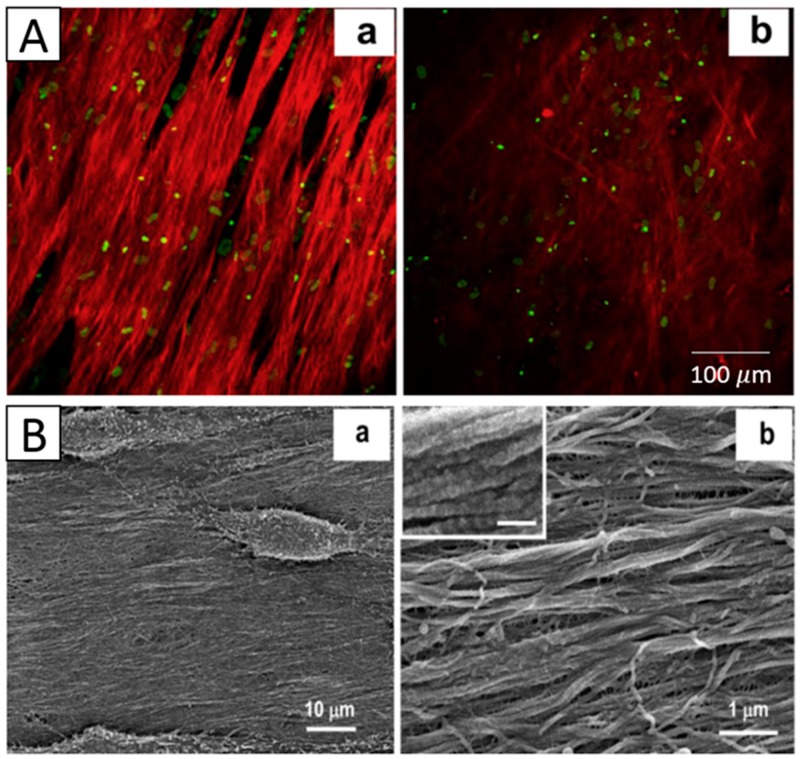
(**A**) Two-photon images of hCSSCs-secreted ECM varying with: (**a**) aligned fibrous, (**b**) random fibrous PEUU mat and (**B**) SEMs of hCSSCs and the elaborated ECM on the (**a**,**b**) aligned fibrous. Reproduced from [8], with permission from © 2012 Elsevier.

Shweta Sharma et al. electrospun PCL nanofibrous scaffold and inoculated limbus epithelial cells on the surface of the scaffold. The results showed that cells infiltrated the nanofibers and successfully formed a 3D corneal epithelium, which was viable for two weeks [14]. Alireza Baradaran-Rafii utilized electrospun a PHBV/gelatin scaffold to culture limbus epithelial cells, and the corneal epithelium also formed [42]. The electrospun silk scaffold and PLGA nanofibrous scaffold were also demonstrated to be suitable substitutes for promoting epithelial cells to form epithelium [47,51]. Jian Wu et al. electrospun an aligned PEUU mat inoculated with hCSSCs, and they found that the PEUU substrate could induce the alignment of hCSSCs, which elaborated a dense collagenous matrix, 8–10 um in thickness, deposited on the PEUU substratum. This matrix contained collagen fibrils with uniform diameter and regular interfibrillar spacing, exhibiting global parallel alignment similar to that of native stroma [8] (Figure 8).

## 5. Conclusions and Future Perspectives

Recently, because of the increasing population with corneal disease and the short-comings of donated fresh cornea, many researchers have devoted themselves to the field of corneal tissue engineering and have already achieved extensive advancements. Although there are still many challenges for the regeneration of full-thickness corneal tissues, the problems that we need to focus on are much clearer than they were a decade ago. The primary work is the fabrication of scaffolds that can mimic the ECM found in native cornea. For this purpose, various methods and materials have been tested for their performance in corneal tissue engineering. Among these methods, the technique of electrospinning has been proven as an effective approach and has been widely studied in recent years. The scaffold fabricated by electrospinning has a high volume-to–surface area ratio and porosity, which simulates natural ECM and provides an atmosphere for corneal cells to attach, move, proliferate and differentiate. By integrating the advanced electrospun scaffold design with fast-growing biotechnologies, it is possible to partially or fully overcome the existing challenges of corneal tissue engineering and to make the translation of electrospun-based engineered corneal tissues for clinical applications more realistic.

The electrospun scaffolds have already shown their abilities for engineering corneal tissues in vitro and many studies have developed optimal scaffolds with similar morphology, mechanical properties and transparency to natural corneal engineering. Previous studies have inoculated corneal cells on scaffolds and studied the interaction of cells and scaffolds, and further had in vivo transplantation. However, few studies have studied the corneal tissue repair mechanism after transplanting scaffolds into injured cornea. This is very important and if this issue is addressed, it can serve as a model of the repair mechanism for other tissues in the body.

## Figures and Tables

**Table 1:** The polymers used for fabricating electrospun scaffolds for corneal tissue engineering applications.

Polymer	Solvent	Concentration	Fiber Diameter	Cell Type	Advantages	Advanced Properties	Ref.
Single polymer							
PCL	TFE	10% *w*/*v*	90–174 nm	Limbal epithelial cell	Biocompatible, able to retain a normal corneal phenotype, promote corneal epithelium formation		[14]
	TFE	10% *w*/*v*	108–172 nm	Human corneal epithelial cell	Bioactive and biocompatible, improved cell attachment	Functionalized by He/O_2_ plasma	[44]
	Chloroform/DMF	10% *w*/*v*	≈310 nm	Rabbit keratocytes	Promote cell attachment and proliferation		[45]
	Chloroform/DMF	5% *w*/*v*	400–800 nm	Rabbit limbal stem cells	Improve mechanical properties, cell attachment and proliferation	Functionalized by plasma	[46]
PLDLA	Chloroform/DMF	2% *w*/*v*		Human corneal stromal cells	Biocompatible, promote reverting corneal fibroblasts to a keratocyte phenotype	Orthogonal multilayers, aligned fibers for each layer	[1]
PLGA	Dichloromethane	25% *w*/*v*	40–130 nm	Rabbit limbal fibroblasts and rabbit limbal epithelial cells	FDA-approved and artificial bionic limbus	Combined with microstereolithography	[27]
	Dichloromethane	25% *w*/*v*		Rabbit limbal epithelial cells	Biocompatible, promote multilayer formation of cells		[47]
PHBV	Chloroform/DMF	10% *w*/*v*	≈1350 nm	Rabbit keratocytes	Promote cell attachment and proliferation		[45]
PEUU	HFIP	5% *w*/*v*	100–220 nm	Human corneal stromal stem cells	Promote the differentiation of stem cells to keratocytes and production of collagen matrix	Aligned fibers	[8]
Collagen	HFIP/DMF	9% *w*/*v*		Retinal pigment epithelium cells and human corneal keratocytes	Suitable for cell attachment and growth and more ECM deposition	High transparency	[48]
	Acetic acid	4%–7.5% *w*/*v*	50–451 nm	Rabbit corneal fibroblasts	Biocompatible, reduced myofibroblast phenotype expression on aligned scaffold	Aligned fibers	[49]
	Acetic acid	4%–7.5% *w*/*v*	160–240 nm	Rabbit corneal fibroblasts	Suitable for cell attachment and growth	Aligned fibers	[50]
Silk	TFE	2.5% *w*/*v*		Human limbal stem cells	Biocompatible, promote corneal epithelium formation	Aligned fibers	[51]
Gelatin	Glacial acetic acid/ethylacetate/distilled water	10% *w*/*v*	60–148 nm		Improved mechanical properties	Aligned fiber-alginate gel and improved transparency	[52]
Blended polymer							
PHBV/Gelatin	TFE	50% *w*/*v*	≈100 nm	Limbal stem cell	Biocompatible, promote cell attachment and proliferation and corneal epithelium formation	Improved transparency	[42]
PGS/PCL	Chloroform/ethanol	13% *w*/*v*	300–550 nm	Human corneal epithelial cell	Increased moduli	Aligned fibers	[53]
Collagen/HA/PEO	Acetic acid	10% *w*/*v*	51.3–106.9 nm	Epithelial cells, fibroblasts	Excellent biocompatibility and mechanical properties, promote cell attachment and corneal epithelium regeneration	Chitosan surface modified and improved transparency	[43]
Gelatin/PLLA	HFIP/DMF	5% *w*/*v*	800–1000 nm	Corneal epithelial cells and keratocytes	Biocompatible, improved mechanical properties	Aligned fibers	[54]
	HFIP/DMF	10% *w*/*v*	750–1000 nm		Improve the regeneration of corneal stroma	Aligned fibers and improved transparency	[55]
SF/P(LLA-CL)	HFIP	8% *w*/*v*	123–649 nm	Human corneal endothelial cells	Promote mechanical properties, cell attachment and proliferation	Improved transparency	[56]
